# Eyes Matched to the Prize: The State of Matched Filters in Insect Visual Circuits

**DOI:** 10.3389/fncir.2018.00026

**Published:** 2018-04-04

**Authors:** Jessica R. Kohn, Sarah L. Heath, Rudy Behnia

**Affiliations:** Department of Neuroscience, Columbia University, New York, NY, United States

**Keywords:** insects, neural circuit, vision, Drosophila, neuromodulation, sensory circuit

## Abstract

Confronted with an ever-changing visual landscape, animals must be able to detect relevant stimuli and translate this information into behavioral output. A visual scene contains an abundance of information: to interpret the entirety of it would be uneconomical. To optimally perform this task, neural mechanisms exist to enhance the detection of important features of the sensory environment while simultaneously filtering out irrelevant information. This can be accomplished by using a circuit design that implements specific “matched filters” that are tuned to relevant stimuli. Following this rule, the well-characterized visual systems of insects have evolved to streamline feature extraction on both a structural and functional level. Here, we review examples of specialized visual microcircuits for vital behaviors across insect species, including feature detection, escape, and estimation of self-motion. Additionally, we discuss how these microcircuits are modulated to weigh relevant input with respect to different internal and behavioral states.

## Introduction

In order to maximize chances of survival, animals must use relevant sensory information to rapidly inform behavioral responses. While even small brains have considerable computational power, they cannot faithfully encode every feature of the surrounding world. As neuronal computation is energetically expensive, such a feat would require enormous amounts of time and energy, resulting in a dramatic reduction in the efficiency of sensory processing. A single cell might achieve efficient coding by minimizing the number of spikes needed to relay information, thus reducing overall energy consumption (Barlow, 1961). How can this concept be applied to a circuit? One solution to this problem is the selective filtering of relevant information through “matched filters.” The term, borrowed from engineering/signal processing vocabulary, was first applied to sensory processing by Rüdiger Wehner to define cells with specialized processing properties that relay specific, essential information about the sensory world (Wehner, 1987). In his original paper, Wehner writes: “Sensory maps are not neutral photographic images cast on some kind of inner neural screen, but devices shaped by particular selection pressures to preprocess sensory information in a way readily translatable into the necessary motor commands.” Following this logic, we expand the term “matched filters” as a metaphor to include higher-level circuits containing ensembles of neurons which extract crucial components of stimuli while ignoring irrelevant information. These circuits allow for focused preprocessing of sensory information from specific behavioral or environmental paradigms. Because behavioral and environmental contexts are not static, a corollary concept of matched filtering is the need for these filters to be optimized depending on context.

Among sensory stimuli, visual input contains an overwhelming quantity of information. The problem of efficient information processing is particularly critical for many insect species, which have small brains and often rely heavily on visual input to survive. As a consequence, insects have evolved to employ a variety of strategies to accomplish visual matched filtering. These strategies are evident at both the structural and functional levels. The abundant structural features specific to the visual systems of the species in question have been extensively reviewed, in particular at the level of the eye and photoreceptors themselves (Warrant, 2016a,b). Here, we expand on visual matched filters by including higher level circuit motifs in insects that are involved in filtering stimuli relevant for particular behaviors.

We discuss insect visual circuit specializations primarily in the fruit fly and the blowfly, and focus on circuits that underlie behaviors necessary for survival: hunting, mating, escape, and flight control. Additionally, we review recent work that has revealed mechanisms in the fruit fly *Drosophila* for ignoring irrelevant self-motion signals while encoding optic flow. Finally, we will explore several examples of how insects achieve a higher degree of specificity in visual microcircuits via neuromodulation.

## Visual circuits in insects are specific and efficient

Despite their small brain size, insects perform a vast repertoire of behaviors necessary for survival. The efficiency of executing these tasks is increased by visual microcircuits that are designed to propagate behaviorally relevant stimuli while filtering out irrelevant information. In the following sections, we discuss insect visual matched filters for informing behavior.

### Matched filters in target tracking and feature detection

In order to successfully mate and hunt for prey, insects must distinguish targets from the surrounding environment before they can engage in rapid pursuit. Dragonflies and hoverflies have been studied as models for such target detection, as they exhibit tightly controlled pursuit behavior of small targets (Collett and Land, 1975; Olberg et al., 2007; Olberg, 2012). In these insects, small target motion detectors (STMDs) have been identified electrophysiologically in the lobula neuropil of the optic lobe. STMDs are narrowly tuned to objects that comprise 1–3° of the visual field (Collett, 1971; O'Carroll, 1993; Nordström, 2012). It is thought that interactions between neighboring units confer this size selectivity, but details of the circuit mechanisms are still under investigation (Bolzon et al., 2009). In dragonflies, STMDs have small responses to moving light or dark edges. However, when shown a small, dark moving target, STMDs respond supralinearly. That is, their responses to the small moving target are larger than a simple linear combination of their responses to light or dark edges. This indicates that STMDs are specifically tuned to relay information regarding the identification of prey and of conspecifics against the sky (Wiederman et al., 2013). These circuit mechanisms fit the ecological needs of species that hunt in the sky during continuous flight- a strategy known as “hawking.”

However, solutions for detecting dark objects against a bright background do not address target pursuit in a cluttered environment where the contrast between the target and the background is continuously changing. Moreover, how can background *motion*, another confounding variable, be accounted for and filtered out? Wiederman et al. propose a model in which the temporal dynamics of the dark target's leading and trailing edges activate a subset of dragonfly STMDs, regardless of background motion or texture (Wiederman et al., 2013). The filtering out of background motion results in a class of cells that is highly unlikely to be driven by anything but the small target that the animal is tracking. Ultimately, target-tracking information from STMDs is used to control flight steering. Dragonfly STMDs synapse onto a set of neurons known as target selective descending neurons (TSDNs), which project directly to wing motor centers (Olberg, 1981, 1986; Gonzalez-Bellido et al., 2013). STMDs thus serve as matched filters to isolate information about small moving targets, and relay this information to rapidly inform behavioral responses.

Feature detection becomes an increasingly difficult task when there are low contrast levels in the environment. Insect species that are most active in dim light or in darkness have numerous adaptations that allow them to increase contrast sensitivity to suit their ecological needs. Many of these adaptations occur at the level of photoreceptors (Frederiksen et al., 2008; Honkanen et al., 2017). The hawkmoth *Deilephila elpenor*, however, is nocturnal and implements a circuit mechanism to compensate for extremely low light. These moths must detect moving stimuli in starlight conditions, requiring contrast sensitivity superior to what their retina allows. They accomplish this feat via a specialized circuit designed to sum light both spatially and temporally. This enhances slow, coarse features in the visual scene, and confers sensitivity to light levels 100 times dimmer than without summation (Stockl et al., 2016). The circuit that sums visual inputs in the hawkmoth optic lobe acts as a matched filter to streamline detection of moving targets in starlight. Thus, hawkmoth motion vision has evolved to function optimally in the species' most common state, gaining efficiency at the cost of losing both spatial and temporal resolution.

### Specialized visual circuits in *Drosophila* escape behavior

Escape responses must be executed rapidly to provide the most favorable chances of survival. Thus, escape behavior is an excellent system for examining streamlined circuits that have a robust effect on behavior. Many insects have circuits tuned to detect threatening visual information. For example, the presence of a looming object in the visual field is indicative of an approaching predator, and necessitates a specific, efficient circuit that can inform the best possible escape response. Loom-sensitive pathways are particularly well characterized in *Drosophila*, and act to initiate a motor program for steering away from the potential threat (Gabbiani et al., 2002; Santer et al., 2008).

To escape from a looming predator, *Drosophila* must select one of two escape behaviors: a short-duration escape program that sacrifices flight stability for initial escape speed (von Reyn et al., 2014), and a slower, deliberate escape response that includes flight preparation and wing adjustment (Card and Dickinson, 2008; Fotowat et al., 2009; de Vries and Clandinin, 2012). Both outcomes rely on input from a cell known as the giant fiber (GF) interneuron, which spikes in response to looms, and synapses onto the fly's “jump” muscle to drive an escape response (Tanouye and Wyman, 1980; Allen et al., 2006). The timing of loom-evoked spikes in the GF, relative to input from a proposed alternative descending pathway onto the jump muscle, determines the escape program initiated in the fly (von Reyn et al., 2014). Specifically, the GF interneuron is necessary for short, rapid escape responses, but also informs the slower escape program. In addition, the large diameter of the GF interneuron axon enables a rapid and dependable signal transmission to facilitate a robust escape response (Eaton, 1984).

Detecting looming objects poses a problem, as the local features of looming stimuli are very similar to those of other visual motion. Loom-sensitive inputs to the GF interneuron must remain sensitive to radial motion outward from a central point, which represents a loom, and reject other motion stimuli that look similar at the local level. Recent work by Klapoetke et al. suggests that the lobula plate/lobula columnar type II (LPLC2) cell is a presynaptic partner of the GF interneuron, and provides highly specific information about looming stimuli by nonlinearly integrating inputs from the *Drosophila* motion vision pathway. Specifically, LPLC2s demonstrate “radial opponency”; they are strongly driven by motion outward from the center of their receptive field, and inhibited by inward motion (Klapoetke et al., 2017). The output of LPLC2 cells provides the GF interneuron with information about the angular size of a looming object.

Additional work has shown that the GF interneuron response integrates both the angular size *and* the angular velocity of a looming object. Information about the angular velocity of looming objects is provided by the type 4 lobula columnar (LC4) cell, a second presynaptic partner of the GF interneuron. LC4 is tuned specifically to rapid expansion of a large object profile, and enhances GF responses as looms become more abrupt (von Reyn et al., 2017). By linearly integrating input from LC4 and LPLC2 pathways, GF activity increases the probability of a rapid escape during fast looms. Therefore, parallel representations of looming in *Drosophila* can regulate action selection in a threatening scenario. More importantly, highly specific escape circuits like this one allow the animal to ignore irrelevant motion stimuli and focus on a threatening looming presence. They thus act as matched filters to rapidly initiate an escape response critical for survival.

### Circuits for estimating self-motion

**Figure 1:**
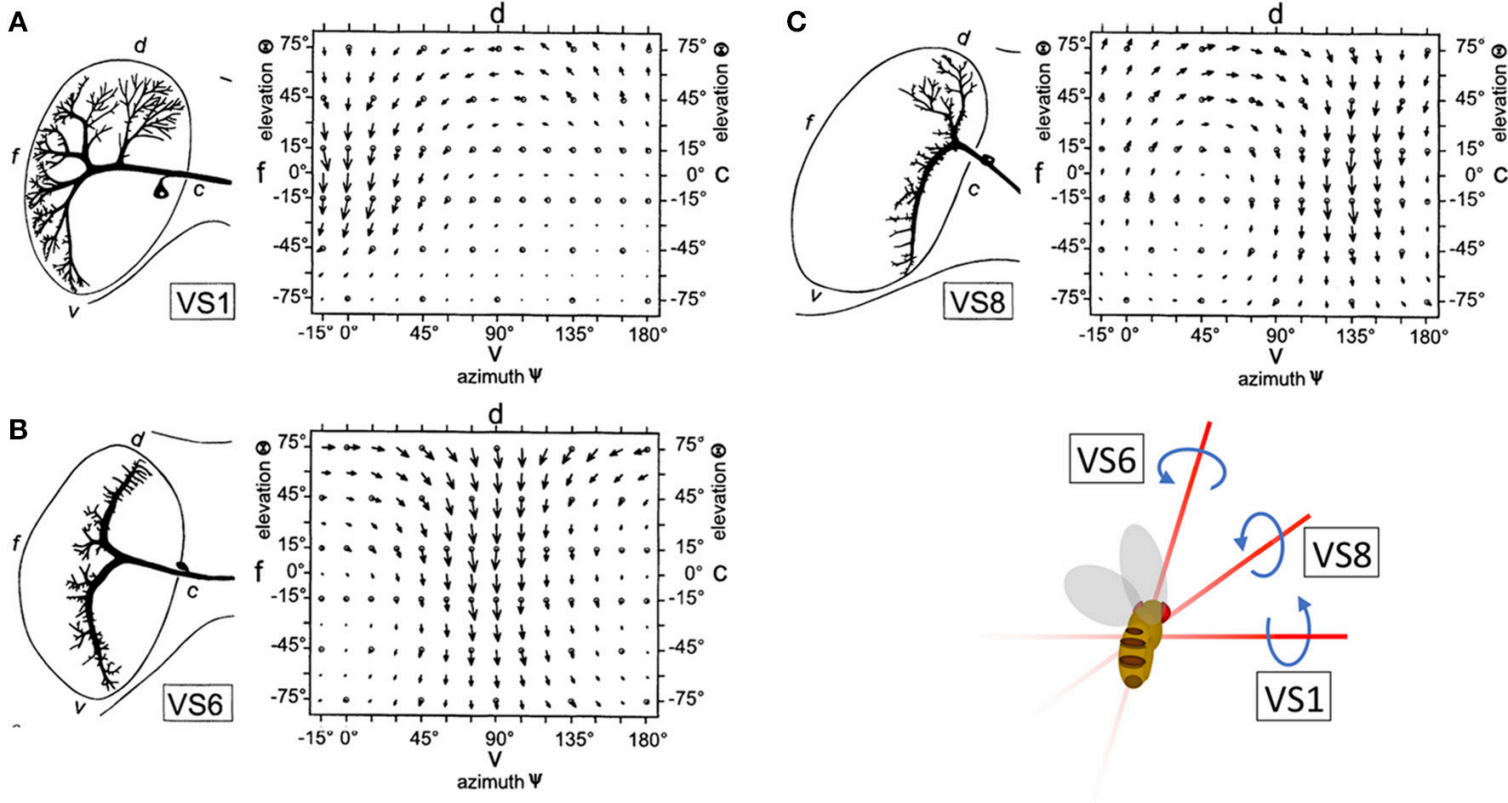
The receptive fields of right hemisphere LPTC cells are tuned to rotational motion. The receptive fields of VS1 **(A)**, VS6 **(B)**, and VS8 **(C)** are tuned to vector fields that correspond to optic flow resulting from a change in pitch, roll, and simultaneous roll and pitch, respectively. Reprinted by permission from Macmillan Publishers Ltd., Copyright (1996), Krapp and Hengstenberg (1996).

To maintain the stability of their flight path, insects must rapidly classify and account for the apparent motion of the visual scene, or optic flow, that results from changes in direction. This task is achieved, in part, by the output of horizontal (HS) and vertical (VS) system cells: wide-field direction sensitive neurons (lobula plate tangential cells, or LPTCs) in the lobula plate of the *Drosophila* optic lobe. HS and VS output is interpreted by downstream circuits to provide gaze stabilization during movement (Hausen, 1982; Strausfeld and Bassemir, 1985; Krapp and Hengstenberg, 1996; Suver et al., 2016). VS and HS preferentially respond to motion in one of the four cardinal directions (up, down, forward, and backward). However, local computations in the dendrites of LPTCs are more nuanced than the global preference of the entire cells. That is, the local directional preferences of LPTCs are consistent with those present in optic flow fields over the entire eye of the animal during natural rotational movements in flight, such as pitch, yaw, and roll (Krapp and Hengstenberg, 1996; Krapp et al., 1998) (Figures 1A–C). For example, the response field of the *Drosophila* VS6 cell matches the flow field corresponding to a roll. Output from VS6 signals a roll to downstream circuits, which drives corrective motion by providing compensatory signals to neck muscles to return the head to its original position. This is an example of a matched filter that increases efficiency in motion correction by relaying information about rotational movement.

VS and HS cells, which respond to natural *rotational* movements, are perhaps the most well characterized higher matched filters in the insect visual system. However, flight control also requires integration of the *translational* motion signals that result from flight along a straight path. Longden et al. recently described a novel set of LPTCs in the blowfly *Calliphora vicina*, known as vertical translation (VT) cells. VTs are tuned to moving background clutter with local receptive fields matched to translational motion in the form of thrust, lift, and sideslip, and fire rapid spike bursts in response to translational motion (Longden et al., 2017). Longden *et al*. hypothesize that these spike bursts allow VTs to encode parallel streams of information, including motion direction, temporal frequency, and parallax (Longden et al., 2017). Due to the precise tuning of VS, HS, and VT cells, one can consider the output of motion detecting circuits in flies to be a well-organized system for flight control that uses matched filters for self-motion to produce path-stabilizing behavioral responses.

### Self-motion signals refine encoding of optic flow

While LPTCs are finely tuned to *detect* relevant stimuli, it is important to consider information that these cells must *ignore* to properly inform behavior. For example, to accurately navigate their surroundings, animals must be able to account for the changes in their visual field that result from their own ongoing movements. As discussed in the previous section, LPTC cells are tuned to optic flow resulting from self-motion, and LPTC output is used by the neck-motor system for gaze stabilization. However, saccades, which are intentional changes in body heading, produce visual information that would normally be interpreted by downstream circuits as an unintentional turn: in this case, a stabilizing response would be counter-productive, since it would act against the intended directional change. While filtering self-motion from LPTC responses is not in and of itself a matched filter, it is an interesting example of how circuit mechanisms are employed to tune a matched filter's output.

Recent studies have elucidated mechanisms by which circuits in the fly optic lobe are able to filter out irrelevant self-motion while preserving responses to pertinent visual signals. A study by Kim et al. showed the first evidence for efference copy in *Drosophila*, describing a suppressive mechanism for visual input in *Drosophila* motion vision circuits, where horizontal system (HS) cells are suppressed by motor-related inputs during saccades in the direction of their sensitivity, despite the lack of a visual input (Kim et al., 2015). Later work by the same group determined that the more a cell was tuned to rotation along a particular axis, the more strongly it was silenced during a flight saccade in order to prevent a corrective head movement. This precise tuning allows for quelling of the corrective yaw signal during intentional turns, while allowing the propagation of visual information along other directional axes to stabilize flight (Kim et al., 2017).

**Figure 2:**
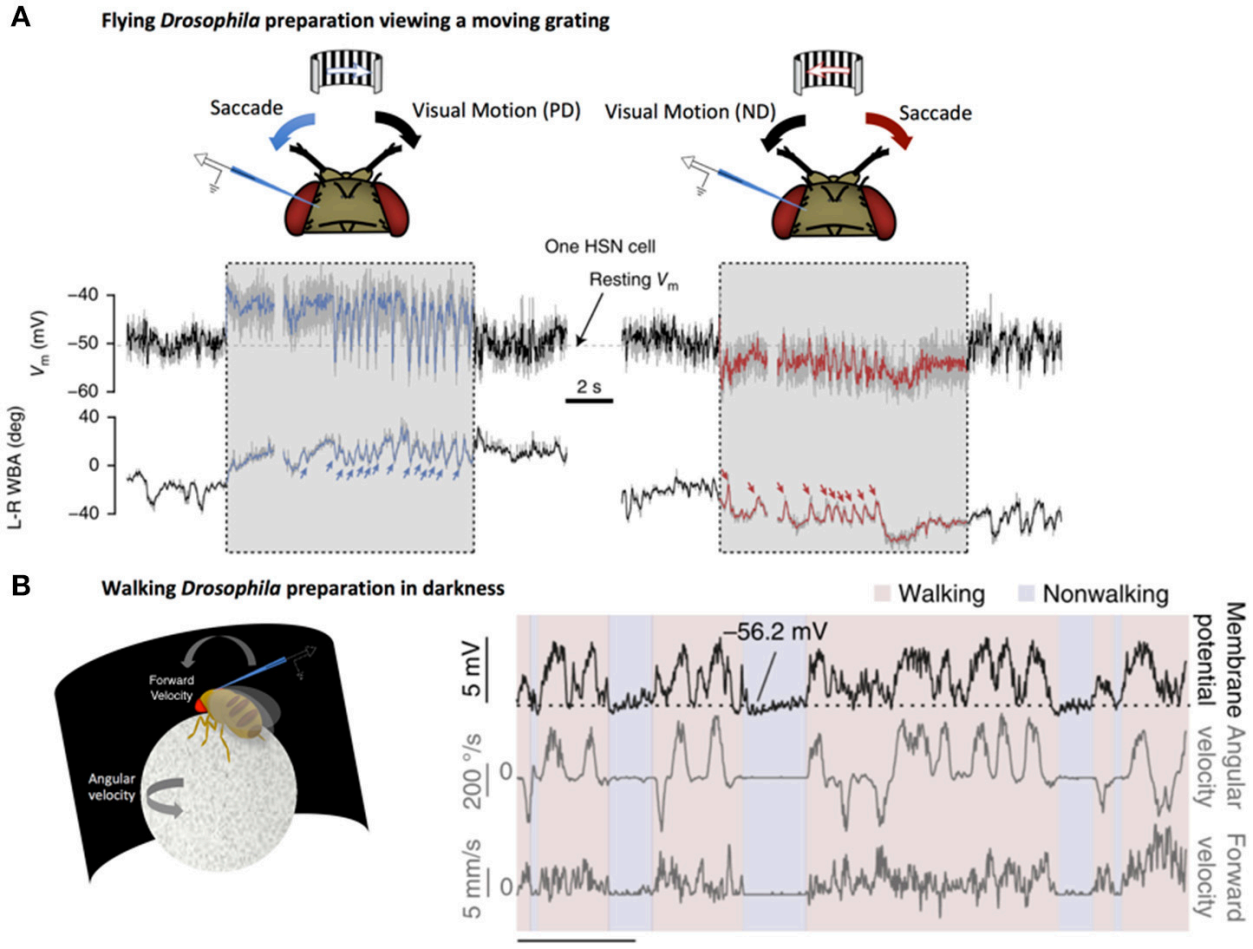
LPTCs receive motor-related input. **(A)** Electrophysiological recording of HS cell activity in a flying *Drosophila* preparation. Saccades, noted by sharp changes in wing-beat amplitude (WBA, lower traces), correspond to rapid fluctuations in HS responses to a moving grating (denoted by the gray box). These saccade related potentials (SRPs) are sufficient to counteract reafferant visual information in the preferred direction (*left*, blue) and the null direction (*right*, red). Reprinted by permission from RightsLink Permissions Springer Customer Service Centre GmbH: Copyright (2015), Kim et al. (2015). **(B)** *Left:* Electrophysiological recording from a right-side *Drosophila* HS cell in darkness during a walking preparation. *Right:* The cell is depolarized (*top trace*) when the fly turns (*middle trace*) in a direction that would, in light, produce optic flow corresponding to preferred-direction motion. Forward velocity (*bottom trace*) does not show the same clear relationship to membrane potential. Reprinted by permission from Macmillan Publishers Ltd: Copyright (2017), Fujiwara et al. (2017).

Further studies have shown that HS cells responses can also be enhanced by behavior-related inputs. By recording from flies walking on a ball, Fujiwara et al. demonstrated that HS cells integrate quantitative non-visual estimates about walking behavior to enhance direction-selective signals from HS (Fujiwara et al., 2017). Importantly, this enhancement only occurs when the direction-selective signals coincide with expected visual outcomes (Figure 2). This selective augmentation is thought to serve as a mechanism to correct for self-motion.

These studies highlight the fact that different behavioral states rely on matched filters to relay varying aspects of visual information: when flies are walking or flying, they must consider visual input that results in changes in direction. In addition to these fast acting compensatory or enhancing mechanisms, state-dependent modulation of visual circuits acts on a longer timescale to allow circuits to adjust their sensitivity to relevant stimuli. In the following section, we focus on these state-dependent changes in the context of matched filters.

## State dependent modulation of visual circuits

In previous sections, we discuss microcircuits that have evolved to efficiently relay pertinent visual information to inform behavioral responses. While a great deal of attention has been given to characterizing the function and anatomy of these circuits, one of their less studied but equally vital assets is the ability to adapt to different environmental and behavioral states.

### State dependent modulation of tuning

Locomotion causes an increase in the relative speed of the visual scene due to self-motion. This presents a processing problem, as insects must detect increasing speeds of motion between quiescence and flight. Neuromodulation allows visual microcircuits to adjust their sensitivity to different speeds depending on whether an animal is resting, walking, or flying: microcircuits shift their peak sensitivity to faster or slower motion depending on behavioral state (O'Carroll et al., 1996; Joesch et al., 2008; Chiappe et al., 2010; Arenz et al., 2017). Accordingly, insect circuits for motion detection can be thought of as anatomically rigid microcircuits designed to filter relevant motion within a small range, with neuromodulation providing an additional layer of state-dependent flexibility.

**Figure 3:**
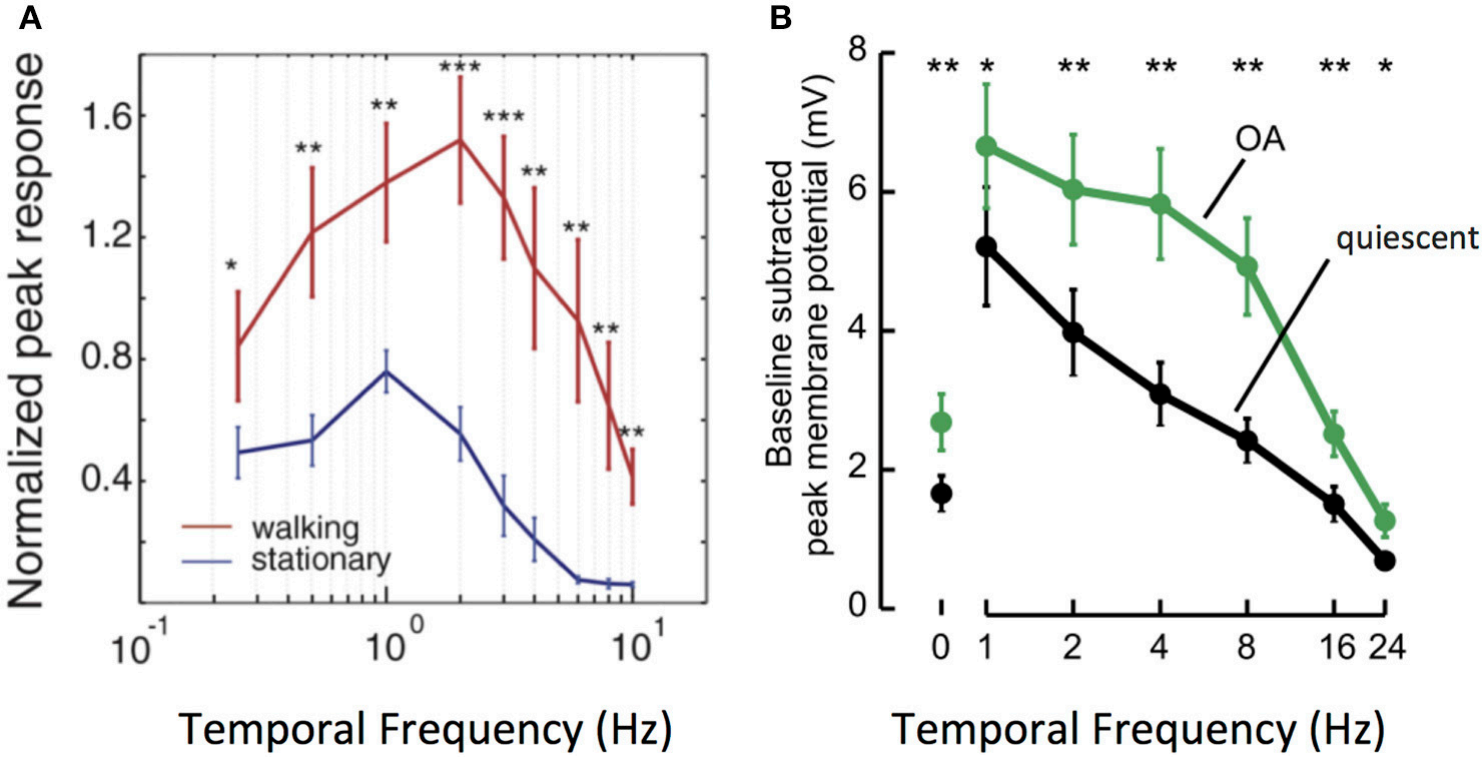
Locomotion affects the gain and tuning of LPTC cells. **(A)** Tuning curves of horizontal system (HS) LPTC cells in *Drosophila* during quiescence and walking. During walking, [Ca2+] responses increase in amplitude, and peak responses shift to faster levels of motion. Reprinted from Current Biology, 20, Chiappe et al. (2010). Walking modulates speed sensitivity in *Drosophila* motion vision. Pages 1470–1475, Copyright (2010), with permission from Elsevier. ^*^p < 0.05; ^**^p < 0.01; ^***^p < 0.001. **(B)** Bath Octopamine (OA) application increases the [Ca2+] responses of vertical system (VS) LPTC cells to gratings at faster frequencies, recapitulating the effect of locomotion. Reprinted from Current Biology, 22, Suver et al. (2012) Octopamine neurons mediate flight-induced modulation of visual processing in *Drosophila*. Pages 2294–2302, Copyright (2012), with permission from Elsevier. ^*^, ^**^ Indicate significance at alpha = 0.05, 0.01, respectively.

This phenomenon is particularly well–studied in a number of fly species. An important feature of fly motion vision circuits lies in the sensitivity of their main outputs, LPTCs. As discussed previously, LPTCs are direction selective. The frequency tuning curves of LPTC responses are bell-shaped, with peak sensitivity occurring in response to motion stimuli at approximately 1 Hz in quiescent flies (Joesch et al., 2008; Chiappe et al., 2010; Maimon et al., 2010; Jung et al., 2011; Suver et al., 2012). Work in both fruit flies and blowflies demonstrates that locomotion in the forms of walking and flying modulates the sensitivity of the LPTC tuning curve toward higher frequencies: peak LPTC sensitivity in walking flies occurs in response to 2 Hz stimuli (Chiappe et al., 2010), while LPTC sensitivity in flying flies peaks at 5–10 Hz (Schnell et al., 2014). This corresponds to a shift in circuit sensitivity toward detecting faster motion (Longden and Krapp, 2009; Chiappe et al., 2010; Jung et al., 2011; Schnell et al., 2014) (Figure 3A).

How do motion vision circuits in flies achieve this shift in LPTC sensitivity? The neuromodulator octopamine (OA), the invertebrate correlate of noradrenaline, is released in the optic lobe during locomotion to modulate LPTC tuning (Longden and Krapp, 2009; Suver et al., 2012). Indeed, application of OA or of chlordimeform (CDM, an OA agonist) recapitulates the effects of locomotion on motion vision circuits, as does experimental activation of octopaminergic cells in the optic lobe (Figure 3B). Inhibition of the same cells abolishes the effect seen during flight (Longden and Krapp, 2010; Jung et al., 2011; Suver et al., 2012; Lüders and Kurtz, 2015; Wasserman et al., 2015; Arenz et al., 2017).

In addition to different behavioral states, other environmental changes also require adjustment of the tuning of matched filters. For example, polarized skylight is an important navigational tool for many insect species. However, the pattern of polarization changes with the sun's path across the sky. As solar elevation changes during the day, so do the electric (e)-vector angles of polarized light. This creates a problem for insects that use solar polarization pattern for navigation: without compensating for time of day, matched filters for specific e-vector angles would only function properly when the sun occupies a specific location in the sky. To account for this, the desert locust adjusts the tuning of so-called LoTu1 and TuTu1 neurons in the anterior optic tubercle (AOTu) to detect e-vectors corresponding to the correct solar azimuth at various times in the day. Thus, while these neurons act as matched filters for the e-vectors of polarized light, they must be modulated in order to remain sensitive to relevant information (Pfeiffer and Homberg, 2007). Such an ability to compensate for solar movement has also been noted in in monarch butterflies, bees, and ants (Wehner and Lanfranconi, 1981; Perez et al., 1997; Homberg et al., 2011; Dovey et al., 2013), and allows the insect to remain sensitive to time-dependent relevant information (Giebultowicz, 2000). While the mechanisms underlying such time-dependent tuning have not been defined, they likely involve neuropeptides from the circadian system. Indeed, circadian rhythms modulate numerous insect behaviors including locomotion, flight, feeding, and mating (Giebultowicz, 2000). Future work will determine the role of circadian neuromodulators on the visual microcircuits underlying these behaviors.

### State dependent modulation of gain

Eliciting maximum responses to a particular stimulus at all times is not energy efficient. Thus, some signals must occasionally be assigned greater weight in different behavioral and environmental scenarios. Gain modulation allows insects to maximize the signal to noise ratio of specific visual circuits depending on circumstance. For instance, in addition to shifting the sensitivity of direction selective cells toward faster motion discussed in the previous section, walking and active flight also result in a tonic increase of baseline activity and an increased gain of LPTC responses. This gain increase assigns higher weight to relevant direction signals during locomotion (Longden and Krapp, 2010; Maimon et al., 2010; Suver et al., 2012).

In addition to locomotion, a number of behavioral states call for gain modulation of visual matched filters. One such behavioral state is “odor-tracking,” i.e., following a plume of appetitive odor to locate a food source. In *Drosophila*, odor tracking boosts the gain of Hx, a wide-field interneuron in the lobula plate selective for front-to-back motion (Wasserman et al., 2015). As Wasserman et al. show, this effect is achieved through the activation of octopaminergic neurons with an olfactory stimulus. Thus, the gain of Hx, a matched filter for lateral motion, is modulated in a context dependent manner. Because Hx activity allows *Drosophila* to maintain a stable heading during odor tracking (Chow et al., 2011; Wasserman et al., 2015), increasing the gain of Hx when an odor plume is appetitive increases the likelihood of finding a food source.

In addition to *Drosophila*, a variety of other insect species rely on following odor plumes to find food sources. Mounting evidence indicates that the integration of different sensory modalities is a vital mechanism for modulating visual matched filters to best locate food sources. Female mosquitos, which follow plumes of carbon dioxide to locate food sources, had once been thought to couple olfactory identification of carbon dioxide with thermal detection to hone in on their prey. However, recent work by van Breugel et al. shows that the presence of carbon dioxide is not coupled to thermal detection. Rather, the detection of carbon dioxide increases the gain of visual stimuli representing food sources (van Breugel et al., 2015). This suggests that integrating different sensory modalities is a context-specific manner of modulating relevant matched filters; because similar mechanisms have been noted in various insect species, including *Drosophila* (Wasserman et al., 2015) and even hawkmoths (Raguso and Willis, 2002), such a mechanism is likely evolutionarily conserved.

When considered in the broad context of increasing or decreasing the gain of matched filters, the advantages of modulation in different states becomes clear: rather than eliciting the same level response to a stimulus and sorting through visual information with a low signal-to-noise ratio, insects conserve processing power by increasing the gain of matched filters only in scenarios in which they are important, and suppressing the output of irrelevant matched filters.

However, turning up the gain of a particular response requires extra energy. In situations where metabolic cost cannot be easily restored, such as during starvation, it is no longer advantageous to increase activity within motion vision circuits. As Longden et al. show in the blowfly visual system, the increased activity level of LPTCs seen during locomotion is no longer present in starved animals (Longden et al., 2014). Together, these findings demonstrate that matched filters are not constrained to informing behavioral responses in one single behavioral state, and are modulated in a manner that also considers metabolic cost.

### Arousal modulates gating of visual output

In the context of matched filters, a particularly intriguing scenario involves increasing the specificity of a matched filter following earlier activity. Recent studies have focused on the role of such arousal-mediated mechanisms in enhancing the responses of specific microcircuits in a state-dependent manner.

**Figure 4:**
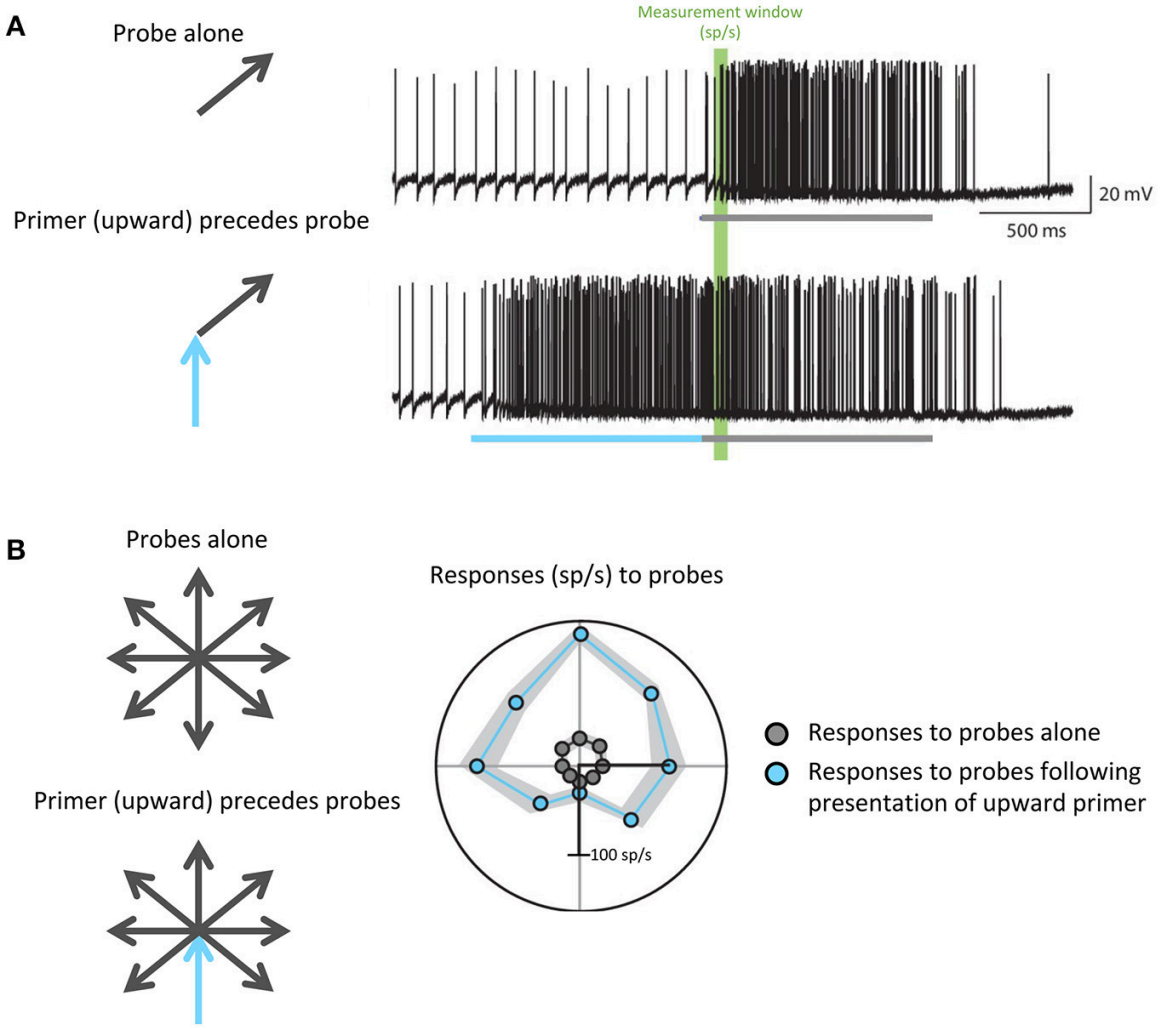
Predictive gain from a primer boosts expected responses and inhibits responses to motion in the opposite direction. **(A)** Electrophysiological recordings from the dragonfly CSTMD1 cell. The top trace shows responses to a single probe (gray) while the bottom trace shows responses to a probe (gray) preceded by a primer (blue) in a similar direction. The portions of the traces highlighted in green indicate the measurement window from which firing rate in spikes per second (sp/s) was calculated. **(B)** Responses to all possible probe directions gray arrows), calculated as the spike rate within the green window in **(A)**, are plotted in gray. In the case of responses to probes that have been preceded by an upward primer (blue arrow), which are plotted in blue, more facilitation occurs in directions similar to that of the primer. Wiederman et al. (2017) eLife 6, 1–19 (2017).

For example, to hunt, dragonflies must accurately track the movement of small targets against a moving background. Dragonflies accomplish this task with the CSTMD1 cell: a matched filter that selectively propagates information about single, small moving objects. Motion in a particular direction modulates the gain of specific areas within the CSTMD1 receptive field to prime its responses for continued motion in the same direction: that is, if the CSTMD1 cell responds to a small object moving in one direction, its subsequent responses to motion along that vector will be increased (Rind et al., 2008; Wiederman et al., 2017). This enhances the predictive ability of CSTMD1, and minimizes future processing time in a task that already requires rapid response (Figure 4). Further studies have revealed attention-like mechanisms in visual processing of *Drosophila* and bumblebees (Morawetz and Spaethe, 2012; Nityananda and Chittka, 2015), indicating that recent visual experience may serve as an efficient and specific mechanism for modulating the gain and specificity of matched filters in relevant behavioral and environmental states.

Similarly, arousal modulates the activity of locust descending contralateral movement detector (DCMD) neurons, and determines whether collision avoidance behavioral programs are initiated. In this case, increasing the arousal level of the animal via mechanical stimulation or by inducing flight primes DCMD cells to switch from a habituated spiking state to a high-frequency response state that mediates evasive maneuvers in flying animals (Rind et al., 2008).

## Conclusions

Even with a limited number of neurons, insects demonstrate incredible versatility in the number of behaviors they produce and the efficiency with which they produce them. To produce these behaviors in a streamlined manner, many insect species rely on matched filters to quickly process only the most important information in a particular scenario. Indeed, matched filtering has proven an excellent evolutionary strategy for reducing the overwhelming amount of information in visual scenes down to a small number of relevant outputs.

As we have discussed in this review, several clear examples of matched filters can be seen in microcircuits used for feature detection, escape, and flight control. These microcircuits filter extraneous information from visual scenes, both to increase the speed with which the animal can perform the necessary behavior and to reduce unnecessary energy consumption.

Rapidly changing environments require these microcircuits to be sensitive to a wide range of stimuli. However, to create a matched filter for every possible variation of a particular stimulus would negate the energy efficiency inherent to matched filters. This problem is solved by the state-dependent modulation of matched filters; a phenomenon that is particularly well studied in the sensitivity of fly LPTC cells to faster motion during locomotion. We highlight this example, as well as state dependent modulation of gain, which lends increased weight to the outputs of circuits relaying relevant behavioral stimuli when metabolic conditions allow for it. The precise mechanisms underlying neuromodulation in these circuits remain poorly understood, especially considering that the same neuromodulator may induce different effects through multiple receptor types or in combination with other neuromodulators (Marder, 2012). Future studies geared toward understanding these mechanisms will shed light on how matched filters are designed to be flexible in a state dependent manner.

A broad theme across the field of matched filters is the balance between weighting important stimuli for informing behavior and conserving energy. This becomes apparent when considering the evolutionary context of a particular microcircuit and the circumstances in which it is modulated. While we discuss only a few examples in insect visual systems in this review, this theme likely extends across sensory systems in many species. Thus, considering specialized circuits as matched filters lends organization into the classification of complicated microcircuits and their state-dependent function.

## Author contributions

All authors listed have made a substantial, direct and intellectual contribution to the work, and approved it for publication.

### Conflict of interest statement

The authors declare that the research was conducted in the absence of any commercial or financial relationships that could be construed as a potential conflict of interest. The handling Editor declared a shared affiliation, though no other collaboration with the authors.
